# A Holistic Approach for Physiotherapy Rehabilitation of Girdlestone Arthroplasty With Infection and Concomitant Contralateral Spastic Hemiplegic Cerebral Palsy: A Case Report

**DOI:** 10.7759/cureus.57689

**Published:** 2024-04-05

**Authors:** Sojwal P Nandanwar, Swapnil U Ramteke

**Affiliations:** 1 Department of Sports Physiotherapy, Ravi Nair Physiotherapy College, Datta Meghe Institute of Higher Education and Research, Wardha, IND

**Keywords:** case report, bimanual hand-arm and lower limb training with mirror therapy for spasticity, cognitive behavioral therapy, physiotherapy, spastic hemiplegic cerebral palsy, girdlestone procedure, femoral head dislocation, hip osteomyelitis

## Abstract

Girdlestone arthroplasty is a traditional approach for complicated infections occurring with contralateral spastic hemiplegic cerebral palsy, which presents intricate challenges in rehabilitation. In this case report, an 18-year-old girl came to a multispecialty hospital with a history of falls. She was an identified case of femoral head dislocation with acute osteomyelitis and a history of spastic hemiplegic cerebral palsy. She underwent girdlestone arthroplasty with additional upper tibial and ankle pin traction. After that, she was referred to physiotherapy management. To further aid recovery, rehabilitation protocol included a combination of static exercises, ankle pumps on the affected side, and stretching, bimanual hand-arm training with lower limb training on the unaffected side to reduce spasticity. Once the stitches were removed and traction discontinued, the focus shifted to improving mobility through basic activities like rolling and transitioning to sitting, gradually progressing to standing with the assistance of a walker and bimanual hand-arm training with lower limb training for spasticity. Outcome measures like functional independence measure, numerical pain rating scale, range of motion, and manual ability classification system were used to record patient progress during rehabilitation. This case report serves the crucial role physiotherapy plays in the treatment of orthopedic and neurological conditions in younger patients, with the ultimate goal of regaining functional independence and enhancing overall quality of life.

## Introduction

Traumatic hip dislocation is rare because the hip joint is highly stable. Hip osteomyelitis is a bone infection in the hip joint, typically caused by bacteria [1]. An uncommon condition that affects 1.3 out of 1,000 adults, femoral head dislocation is frequently associated with hip traumatic injury [1]. Acute osteomyelitis combined with hip dislocation is a serious disorder that causes the femoral head to dislocate from the acetabulum and causes an acute bone infection [2]. When acute osteomyelitis occurs, Staphylococcus aureus is the main cause of subperiosteal abscess, especially in adults and children [3]. Inadequate therapy or bacterial inoculation can cause it directly, which appears as a radiolucent shadow on x-rays [4]. Classification of Cierny-Mader type III osteomyelitis is a localized lesion with medullary and cortical involvement that is mechanically stable following debridement [5]. A classic approach to infections of the acetabular cavity and femoral head is Girdlestone pseudoarthroplasty, which accesses the acetabulum and removes the femoral head and neck [6]. However, Girdlestone pseudoarthroplasty has fallen out of favor, impairs hip function, and advanced antimicrobial treatment improves outcomes without sacrificing ambulation [7]. Nevertheless, the girdlestone procedure still has a role in improving the quality of life in non-ambulatory patients with chronic osteomyelitis [8].

Spastic hemiplegic cerebral palsy is a subtype of cerebral palsy causing muscle stiffness and weakness on one side of the body. It is caused by brain damage or abnormalities during birth, causing motor control and coordination issues. Physiotherapy is crucial for managing this condition, aiming to reduce spasticity, improve strength, enhance motor skills, provide orthotic support, facilitate gait training, and offer education. Spastic hemiplegic cerebral palsy impairs muscular coordination and balance; it enhances the risk of falls [9]. With an incidence of 0.6-1 per 1,000 births, it is a prevalent cause of impairment in children. The primary cause of the majority of children's difficulties with bimanual daily living chores is the paretic hand's poor function, which increases the use of the non-paretic hand in a variety of bimanual tasks [10].

Hip dislocation and spastic hemiplegic cerebral palsy are two conditions that require the integration of physical therapy. Following a fall, people with cerebral palsy may experience hip-related problems such as dislocation of the femur head and osteomyelitis [11]. It seeks to enhance muscle tone, strength, and range of motion, boosting functional capacities. Proprioceptive neuromuscular facilitation, stretching methods, and therapeutic exercises are utilized to improve motor control and reduce spasticity [12]. Physiotherapy for hip dislocations works to stabilize the joint and strengthen the surrounding muscles [13]. Physiotherapists, psychologists, orthopedic professionals, and guardians must work together to create tailored therapy programs.

## Case presentation

Patient information

An 18-year-old girl presented with a history of falling at home on June 30, 2023, resulting in a right hip injury characterized by immediate swelling, inability to move the right lower limb, and pain while performing movement. She was taken to a government hospital in Akola, where radiological screening revealed right femoral head dislocation. Initially managed conservatively with medications for five months, she was subsequently referred to a multispecialty hospital. She had a known history of left-sided hemiplegic spastic cerebral palsy since 2010, for which she received physiotherapy with significant improvement. She was achieved walking with support/ assistance. On November 29, 2023, she visited a multispecialty hospital in Sawangi with hip pain and deformity presented over the right lower limb. Investigations like radiological screening, bone culture, and bone biopsy were done. She was diagnosed with neglected femoral head dislocation and acute osteomyelitis. She underwent a girdlestone procedure on December 6, 2023, followed by upper tibial pin traction for 45 days of immobilization for limb stabilization and facilitating healing. Rehabilitation was continued through physiotherapy and psychological counseling. Table 1 shows a timeline of the event.

**Table 1:** Sequence of event

Events	Date	Sequences of events
Date of injury	30/06/2023	History of fall
Date of previous hospital visit	30/06/2023	Visited the Government hospital, Akola.
Date of admission	29/11/2023	Visited the AVBRH Hospital, Sawangi.
Date of operation	06/12/2023	Girdlestone arthroplasty
Date of traction application	06/12/2023	Upper tibial pin traction (skeletal traction) was applied. Patient and family education was done.
Date of physiotherapy rehabilitation	07/12/2023	Static exercises for the gluteus, quads, and hamstrings, and stretching on the non-affected side.
Date of suture removal	22/12/2023	Stitches removal was done.
Date of traction removal	11/01/2024	Removal of upper tibial pin traction (skeletal traction).
Date of mobilization in bed	12/01/2024	Rolling, supine to sitting, and bedside sitting.
Date of bed mobilization (out-of-bed)	24/01/2024	Standing with the support of a walker and mild assistance.

Clinical finding

After obtaining prior consent from the patient, she was examined and treated.

*On Observation and* *Palpation*

The patient has a mesomorphic build and a body mass index of 20.1 kilograms per square meter. Her temperature was afebrile, with a pulse rate of 80 beats per minute, respiratory rate of 16 breaths per minute, and blood pressure of 122/84 millimeters of mercury. Distal pulses were palpable, which indicated no vascular insufficiency. She was observed supine lying. The right hip and knee were extended, and the ankle was plantarflexed. The left hip was kept in slight flexion, abduction, and internal rotation, with the knee displaying a fixed flexion deformity of 10 degrees, and the left ankle was plantarflexed.

On Examination

Musculoskeletal system: On postoperative day 2, the patient reported significant pain, scoring 8/10 during movement and 5/10 at rest on a numerical pain rating scale. The sutures resulting from the girdlestone procedure measured 13 cm in length and 2 cm in width, with grade 2 tenderness palpated on the lateral aspect of the thigh at the suture site. Due to pain and immobilization, the range of motion for the right hip and knee was not assessed. However, the ankle range of motion was documented (Table 2). Muscle strength assessment using resisted isometric grading of the right hip joint indicated weakness and pain, grade as 4. Segmental limb lengths were measured on the left side from the greater trochanter to the lateral condyle of the femur at 38 cm and from the lateral tibial condyle to the lateral malleolus at 36 cm. Conversely, the right side measured 36 cm from the greater trochanter to the lateral condyle of the femur and 36 cm from the lateral tibial condyle to the lateral malleolus.

**Table 2:** Postoperative assessment of the affected side

Joint movement (Right side)	Active range	Passive range
Ankle dorsiflexion	0-5 degrees	0-10 degrees
Ankle plantarflexion	0-20 degrees	0-30 degrees

Neurological system: The sensory examination of the patient revealed intact sensations. A synergistic pattern was observed in the flexors of both the left upper and lower limbs. According to the Modified Ashworth Scale, the tone was normal on the right side, but on the left side, it was graded as 1 in the upper limb (shoulder, elbow, and wrist), 1+ in the knee joint, and 1 in the lower limb (hip and ankle). Exaggerated reflexes were noted in the left side of the biceps and knee jerks; the planter response showed a positive Babinski's sign on the left side, while other reflexes appeared normal. Range of motion was notably decreased in both the left upper and lower limbs, with the presence of a 10-degree extension lag in the left lower limb. The gait pattern exhibited toe walking and circumductory gait on the left side, accompanied by reduced arm swing. Despite these impairments, the patient demonstrated functional independence in walking, although she required support from stable objects or family members for standing.

Diagnostic investigation

Radiological Finding

**Figure 1:**
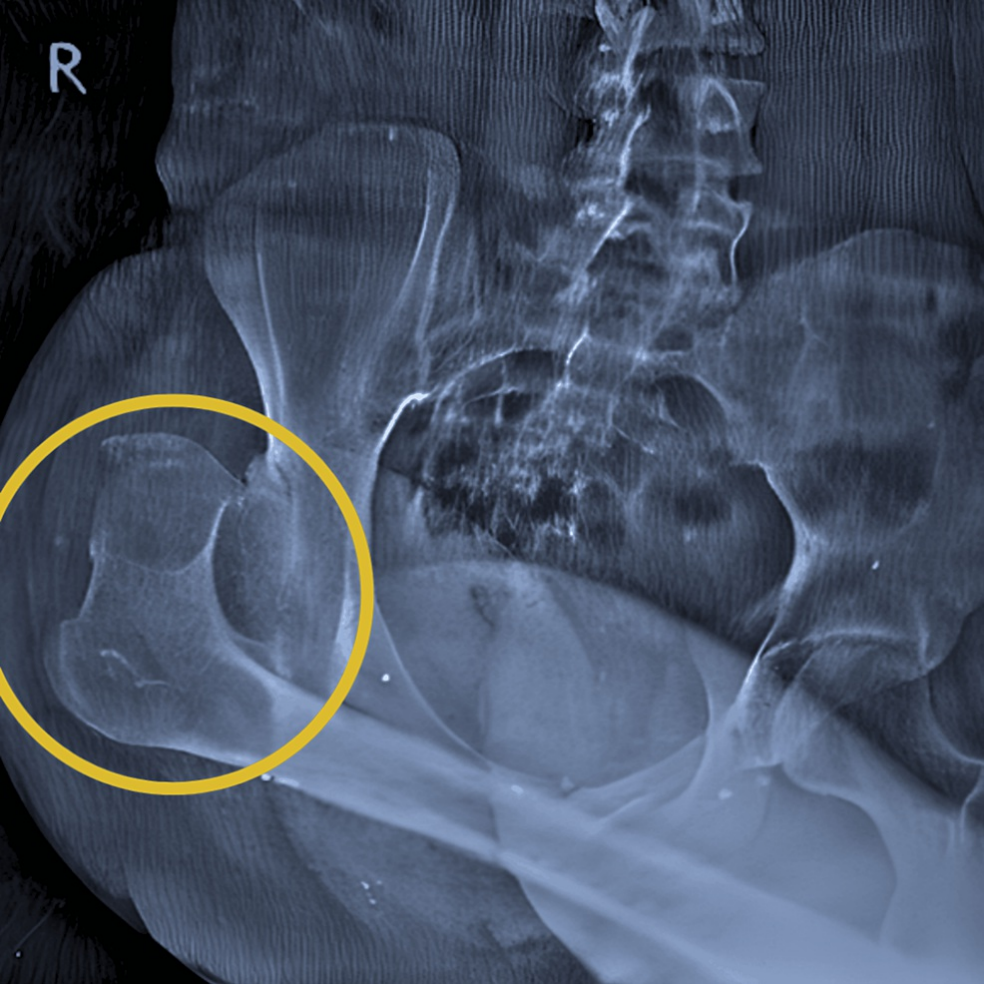
Anteroposterior view of radiograph shows suggestive of right femoral head dislocation The yellow circle shows the dislocation of the right femoral head.

**Figure 2:**
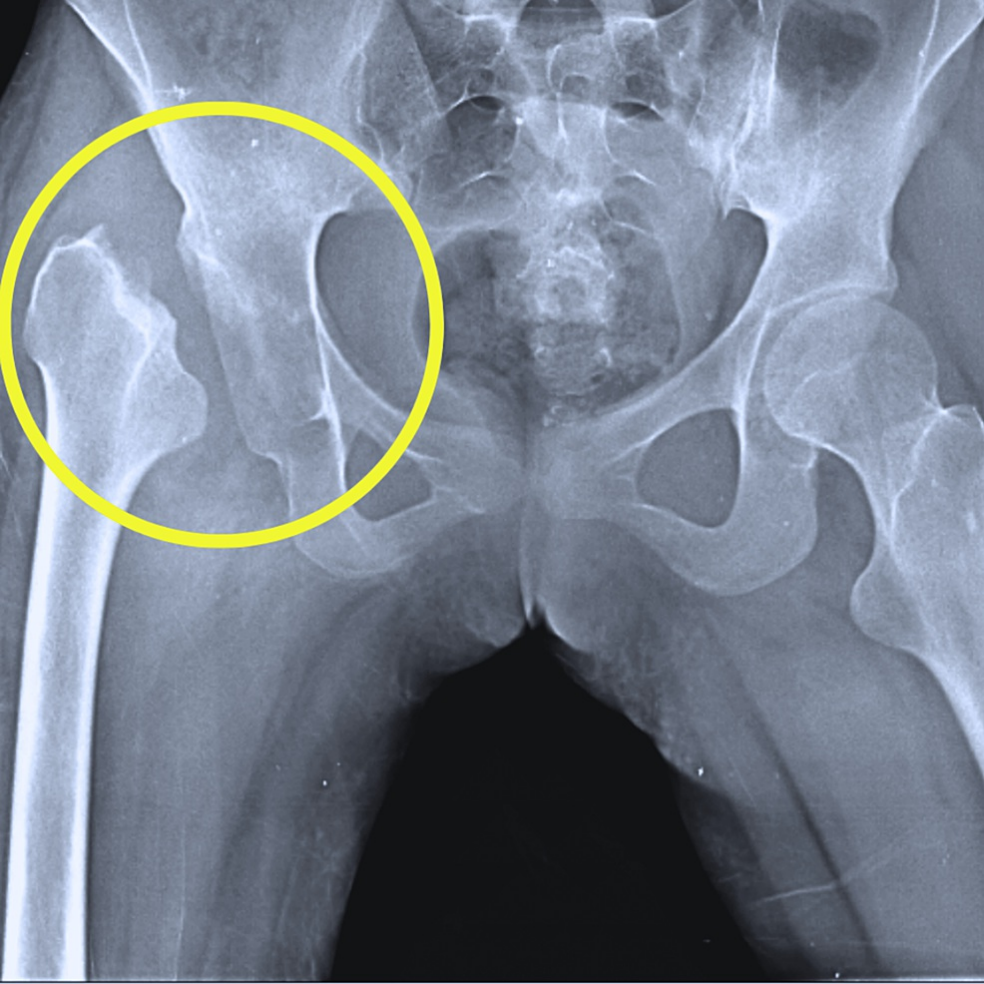
Anteroposterior view of radiograph shows the girdlestone arthroplasty The yellow circle shows the girdlestone arthroplasty.

Pre-operative radiographical screening findings of the pelvis and both hips showed an anteroposterior view suggestive of right femoral head dislocation. Post-operative radiographical screening findings showed that girdlestone arthroplasty. A bone biopsy revealed the presence of inflammatory cells and bone culture positive for S. aureus. Figure 1 shows an anteroposterior view of the radiograph suggestive of right femoral head dislocation. Figure 2 shows an anteroposterior view of the radiograph revealing the girdlestone arthroplasty.

Medical management

Table 3 shows the medical management of the patient.

**Table 3:** Medical management of a patient Tab/ tab: tablet, mg: milligram, tbsp: tablespoon, mcg: micrograms.

Sr.no	Medicine	Generic names	Dosage	Milligram
1	Tab. shelcal	Calcium Carbonate + Vitamin D3	Once daily	1 tab
2	Tab. Nurokind *500*	Methylcobalamin (vitamin b12)	Once daily	1 tab
3	Tab. evion	Levo-carnitine + Vitamin E	Once daily	1 tab
4	Tab. ceftin	Cefixime	Twice daily	200 mg
5	Tab. bexol	Trihexyphenidyl hydrochloride (2 mg)	Thrice daily	2 mg
6	Tab. beclofenac	Beclofenac	Twice daily	20 mg
7	Tab. rubired	Cyanocobalamin 15 mcg +ferrous ascorbate 100 mg+folic acid 1.5 mg+zinc 61.8 mg	Once daily	2 tbsp
8	Tab. limcee	Ascorbic acid (vitamin C)	Twice daily	500 mg
9	Tab. zerodol	Aceclofenac + Paracetamol	See on symptoms	1 tab

Physiotherapy management program

Individualized physiotherapy programs were developed based on the type of abnormalities and the patient's pain level. Weight-bearing was generally discouraged for the first month following surgery, although joint passive and active mobilization was permitted, with special care taken to prevent excessive motions. For the next two months, gradual weight-bearing was permitted until full weight-bearing and the development of an appropriate range of motion were gained. Table 4 shows the patient's week-wise protocol.

**Table 4:** Shows the week-wise protocol of a patient.

Week-wise	Sr. No	Goals	Interventions	Dosage
Day 2- Week 1.5	1.	Patient and family education.	The patient's tolerance and need for treatment vary.	Tell about the recovery period for the condition.
	2.	To reduce anxiety and depression, dysfunctional thoughts	Cognitive behavioral therapy	Sessions were conducted weekly, each session lasting around 1 hour. (For 10 sessions)
	3.	To reduce pain and inflammation.	Cryotherapy (cold pack)	For 10 minutes
	4.	To reduce post-operative circulatory complication	Ankle toe movements and wrist finger movements.	20 repetitions × 2 sets, twice a day.
	5.	To minimize joint stiffness and contractures	Static exercises for gluteus, quadriceps, and hamstrings on both sides.	20 repetitions × 2 sets, twice a day.
	6.	To reduce spasticity of biceps brachii.	Sustained passive stretching of biceps.	30 seconds hold × 3 repetitions
		To improve co-ordination of both arms and functional activities	Hand-arm bimanual training based on motor learning (holding an object and manipulation) with mirror therapy.	1 hour per day.
			PNF techniques D2 flexion to extension pattern to left upper limb (combination of isotonic).	10 repetitions × 1set, twice a day.
	7.	To reduce spasticity of the hamstring, adductors, and tendo-achilles of the left side	Sustained passive stretching of hamstring, adductors, and tendo-achillesof left side.	30 seconds hold × 3 repetitions.
			Slow icing techniques	For 20 minutes.
Week 1.5- 2	1.	To improve mobility and functional independence	Rolling, supine to side lying, and long sitting.	5 repetitions × 3 sets.
	2.	To decrease pain	Cryotherapy	For 10 minutes
	3.	To reduce spasticity of the upper limb	Weight-bearing exercises and weight shift.	20 repetitions × 2 sets, twice a day.
			PNF techniques: Hold-relax technique for elbow joint, then progress to contract-relax (PNF pattern).	10 repetitions × 1set, twice a day.
	4.	To reduce spasticity of the hamstring, adductors, and tendo-achilles of the left side.	Sustained passive stretching of hamstring, adductors, and tendo-achillesof left side.	30 seconds hold × 3 repetitions
	5.	To improve joint range of motion and muscle strength.	Gentle heel sides and ankle toe movements, and continue with static exercises.	10 repetitions × 2 sets
Week 2-4	1.	To improve mobility and functional independence	Side-lying to sitting.	5 repetitions × 1 set
			Bedside sitting.	For 5-10 minutes
	2.	To reduce joint range of motion	Gentle passive movement at the hip joint was initiated. And active range of motion in the knee and ankle joints.	10 repetitions × 1 set.
	3.	To increase muscle strength	Gentle resistance to knee and ankle joints.	10 repetitions × 1 set.
	4.	To reduce spasticity of the upper limb	Weight-bearing exercises and weight shift, peck board activity.	20 repetitions × 2 sets, twice a day.
	5.	To reduce spasticity of the lower limb	"Kick out" activity in high sitting.	10 repetitions × 1 set.
Week 4-6	1.	To improve mobility and functional independence	Sit-to-stand activity and standing exercises.	5 repetitions × 1 set.
	2.	To reduce joint contracture	Relaxed passive stretching of hip flexion, extension, and abduction movement	5-second holds with 10 repetitions.
	3.	To increase the range of motion and joint mobilization	Suspension therapy for the hip joint.	10 repetitions × 1 set.
	4.	To improve joint range of motion	The passive range of motion at the hip joint was upto 30-50 degrees, and active assisted range of motion exercises to the knee joints.	10 repetitions × 1set.
	5.	To increase muscle strength	Resisted active movement of knee and ankle joints.	10 repetitions × 1set.
	6.	To reduce spasticity of the upper limb and lower limb	Functional activities (reaching, grasping)	10 repetitions × 1set.
Week 6 onwards	1.	To improve mobility and functional independence	Standing with support.	10 minutes
	2.	To increase joint range of motion and muscle strength	Suspension therapy for hip joints above 50 degrees. Resisted active movement of knee and ankle joints.	10 repetitions × 1set.
	3.	To reduce spasticity of the upper limb and lower limb	Weight-bearing exercises and weight shift.	10 repetitions × 1set.
	4.	To improve gait training and co-ordination exercises	Partial weight bearing on the affected leg with the help of a walker and mild assistance. According to the patient's progress, the gait training should be modified. (Parallel bar walking) Continue gait training without the patient department.	2-3 rounds of 3-4 large tiles or city blocks.

Outcome measure and follow-up

A weekly physical therapy interventional program was started. Evaluations of the patient both before and after treatment were carried out to track their improvement. Outcome measure results are shown in Table 5.

**Table 5:** Outcome measure

Outcome measures	Day 1	Week 3	Week 6
Functional Independence Measure	28/126	54/126	86/126
Numerical Pain Rating Scale	9/10	2/10	0/10
Manual Ability Classification System	Level 2	Level 1	Level 1

## Discussion

An 18-year-old girl underwent girdlestone arthroplasty for femoral head dislocation with acute osteomyelitis, underscoring the importance of prompt infection control in weight-bearing joints to avoid complications. She was a known case of spastic hemiplegic cerebral palsy. Cognitive behavioral therapy is a treatment method for individuals with cerebral palsy, primarily focusing on psychological targets, with most studies reporting improvement in symptoms post-cognitive behavioral therapy [14]. For the treatment of hip dislocations and spastic hemiplegic cerebral palsy, physiotherapy is an essential tool. It aims to enhance functional abilities, muscle tone, strength, and range of motion [15]. Interventions include proprioceptive neuromuscular facilitation (PNF), strengthening surrounding muscles, increasing the range of motion, stabilizing the hip joint, and employing therapeutic techniques, including ultrasound, electrical stimulation, heat therapy, or cold therapy [16]. According to Waghe et al., a safe return to everyday activities has been made possible by physiotherapeutic intervention, which combines therapeutic exercises, manual techniques, and patient education. It has significantly improved pain management, range of motion, muscular strength, gait, and the prevention of secondary impairments [17]. Using stretching exercises, posture strategies, and neuromuscular re-education by the physical therapist aims to lessen spasticity in patients with spastic hemiplegic cerebral palsy. Passive, active, and dynamic stretching techniques are employed along with therapeutic activities aimed at enhancing motor control, balance, and coordination. In order to improve gait mechanics and enhance independence and quality of life, functional training is integrated into therapy sessions [18]. Hand-Arm Bimanual Intensive Training (HABIT) is a promising intervention for children with hemiplegic cerebral palsy, enhancing bimanual coordination and improving upper limb function. However, challenges like resource-intensive nature and variable treatment responses highlight the need for ongoing research to optimize implementation and identify factors influencing its efficacy. Refinement of HABIT protocols holds the potential to significantly improve the quality of life for these children [19]. Task-oriented training is more effective in improving mobility and balance in spastic cerebral palsy patients compared to proprioceptive neuromuscular facilitation [20].

The case study emphasizes how crucial physical therapy is for enhancing functional mobility and ambulation in patients with persistent osteomyelitis undergoing curettage and debridement. This underappreciated treatment strategy highlights the vital role that rehabilitation plays in improving day-to-day activities and may be especially helpful for people who have experienced a hip dislocation.

## Conclusions

An 18-year-old girl with hip osteomyelitis, femoral head dislocation, and spastic hemiplegic cerebral palsy highlighted the transformative impact of a multidisciplinary approach and tailored rehabilitation strategies by leveraging physiotherapy, mirror therapy, bimanual training, and cognitive behavioral therapy for her recovery. Despite the complex intricacies of her condition and the challenges presented by the girdlestone procedure, the patient experienced notable enhancements in mobility, a reduction in pain, and an increase in functional independence. The diligent tracking of outcome measures highlights the importance of objective evaluation in measuring progress and maximizing patient outcomes. This case underscores the profound impact of collaborative care and comprehensive interventions in facilitating significant improvements in the lives of individuals grappling with complex orthopedic and neurological conditions.
